# Theoretical model of recovery following a suicidal episode (COURAGE): scoping review and narrative synthesis

**DOI:** 10.1192/bjo.2022.599

**Published:** 2022-11-17

**Authors:** Yosef Sokol, Chynna Levin, Mairav Linzer, Chayim Rosensweig, Shifra Hubner, Molly Gromatsky, Samantha Walsh, Lisa Dixon, Marianne Goodman

**Affiliations:** Department of Psychiatry, Icahn School of Medicine at Mount Sinai, New York, New York, USA; Department of Psychology, School of Health Sciences, Touro University, New York, New York, USA; and VISN 2 Mental Illness Research, Education and Clinical Center (MIRECC), James J. Peters Veterans Affairs Medical Center, New York, New York, USA; VISN 2 Mental Illness Research, Education and Clinical Center (MIRECC), James J. Peters Veterans Affairs Medical Center, New York, New York, USA; and Teacher's College, Columbia University, New York, New York, USA; VISN 2 Mental Illness Research, Education and Clinical Center (MIRECC), James J. Peters Veterans Affairs Medical Center, New York, New York, USA; and School of Health Professions and Nursing, Long Island University, New York, New York, USA; VISN 2 Mental Illness Research, Education and Clinical Center (MIRECC), James J. Peters Veterans Affairs Medical Center, New York, New York, USA; and Ferkauf Graduate School of Psychology, Yeshiva University, New York, New York, USA; VISN 2 Mental Illness Research, Education and Clinical Center (MIRECC), James J. Peters Veterans Affairs Medical Center, New York, New York, USA; and School of Health Sciences, Touro College, New York, New York, USA; VISN 2 Mental Illness Research, Education and Clinical Center (MIRECC), James J. Peters Veterans Affairs Medical Center, New York, New York, USA; and Department of Psychiatry, Icahn School of Medicine at Mount Sinai, New York, New York, USA; Levy Library, Icahn School of Medicine at Mount Sinai, New York, New York, USA; New York State Psychiatric Institute, New York, New York, USA; Vagelos College of Physicians and Surgeons, Columbia University/New York-Presbyterian, New York, New York, USA

**Keywords:** Suicide, personal recovery, recovery framework, qualitative research, individual psychotherapy

## Abstract

**Background:**

Despite a significant need, there are currently no rigorously developed empirically based models for what personal recovery from a suicidal episode looks like.

**Aims:**

To develop a theoretical model of personal recovery after a suicidal episode, based on a comprehensive literature review and stakeholder feedback.

**Method:**

A scoping review of all empirical studies on this topic was conducted, followed by a thematic analysis to create a preliminary framework. Consultation-based revisions were then made based on feedback from a stakeholder panel to develop the final theoretical model.

**Results:**

The final model comprised seven themes: choosing life, optimising identity, understanding oneself, rediscovering meaning, acceptance, growing connectedness and empowerment (acronym ‘COURAGE’). Although there are some similarities between COURAGE and other models of personal recovery, there are components, such as ‘choosing life’ and ‘understanding oneself’, that are specific to recovery after an acute suicidal episode.

**Conclusions:**

To our knowledge, this is the first study to use a comprehensive literature review with stakeholder feedback to develop a conceptual model of personal recovery after an acute suicidal episode. This model has important implications for both researchers and clinicians to consider. Looking ahead, COURAGE can inform the reconceptualisation of assessment, research and clinical care of individuals who have experienced a suicidal episode.

The traditional medical model of mental health recovery focuses on ‘curing’ clinical symptoms.^1^ A more contemporary conceptualisation has evolved from the development of models of clinical recovery towards models of personal recovery,^2^ which has led to a focus on patient-centred outcomes in applied settings. This has emerged through a deepened understanding of the experiences and needs of people with mental illness. Thus, there has been an increased focus on ‘personal recovery’, including living a fulfilling and rewarding life with positive changes in self-perception and self-experience even with the ongoing presence of mental illness.^2^

In the past few decades, personal recovery evolved as a nuanced construct with multiple overlapping definitions and frameworks. The Substance Abuse and Mental Health Services Administration's (SAMHSA's) working definition of ‘recovery’ from mental illness is ‘a process of change through which individuals improve their health and wellness, live a self-directed life, and strive to reach their full potential’.^3^ Similarly, a review of 89 studies focusing on personal recovery in mental illness identified five overarching processes in an influential recovery framework: connectedness; hope and optimism about the future; identity; meaning in life; and empowerment (acronym ‘CHIME’).^4^

Since the 1980s, progress made in multiple areas of personal recovery theory has influenced the development of recovery-oriented care^5,6^ and personal recovery-focused assessment measures. One well-accepted measure for general personal recovery from mental illness is the Recovery Assessment Scale (RAS).^7^ In addition, there has been a recognition that although there may be general personal recovery theories that apply to all mental illness, there may also be specific personal recovery features based on distinct characteristics of the individual in recovery. Therefore, theoretical models have been developed to target the individual recovery needs of specific populations, such as those with addiction.^8–10^ Researchers have also developed and validated population-specific personal recovery assessment tools. These include measures assessing personal recovery in individuals with schizophrenia (Subjective Recovery Assessment Scale, SubRAS),^11^ the Bipolar Recovery Questionnaire (BRQ),^12^ the Functioning and Recovery Scale^13^ and a trauma recovery assessment called the Solution Focused Recovery Scale (SFRS).^14^

Recovery following a suicidal crisis (post-acute suicidal episode, PASE) is a critical issue for many, as evidenced by current estimates of the prevalence of suicidal thoughts and behaviours. In 2020, approximately 12.2 million adults in the USA considered a suicide attempt seriously, 3.2 planned a suicide attempt and 1.2 million attempted suicide.^15^

Unfortunately, there is currently a critical gap in personal recovery-oriented theory, research and care in the area of suicide. Specifically, there is no model for personal recovery after an acute suicidal episode empirically derived from the suicide literature, let alone one developed using contributions from individuals with lived experience. Possibly owing to the lack of an empirically based and accepted theoretical model for personal recovery for these individuals, there has also been limited work towards developing validated assessment tools and focused research on personal recovery after an acute suicidal episode.

To address the lack of PASE recovery models, this study aimed to (a) conduct a scoping review of the existing literature reporting on PASE recovery framework constructs, (b) identify themes related to PASE recovery characteristics to develop a preliminary framework for a theoretical model of PASE recovery and (c) revise the model based on stakeholder feedback (scientific experts and individuals with lived experience) in this domain.

## Method

Study procedures were informed by the research method used by Leamy and colleagues to develop the CHIME recovery model.^4^ Similar to Leamy et al, we used a scoping review to address our research aim, in accordance with the criteria outlined by Munn et al, differentiating between a scoping and systematic review.^16^ Specifically, their fourth metric for a scoping review, to identify key characteristics or factors related to a concept, was in line with our aim.^16^ Our review adhered to the reporting guidelines of the Preferred Reporting Items for Systematic Reviews and Meta-Analyses extension for scoping reviews (PRISMA-ScR).^17^ Next, qualitative analysis of included article content was completed to develop a preliminary framework. Last, we sought consultation from experts in the field and those with lived experience to create the theoretical model.

### Study identification

**Fig. 1:**
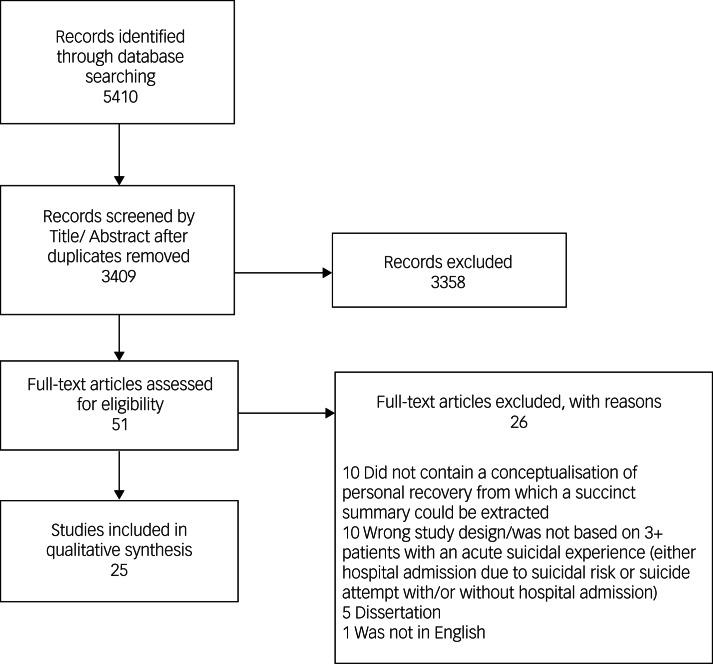
PRISMA flow diagram.

We sought to identify all articles describing underlying components of existing theories, models or explanatory frameworks that depict individuals’ trajectories towards personal recovery after an acute suicidal episode. For the purposes of this search, we defined an acute suicidal episode as including either hospital admission due to risk of suicide or a suicide attempt with/or without hospital admission. We first identified all articles addressing a mental health recovery framework for personal recovery from a suicidal episode, as outlined by Leamy et al.^4^ A comprehensive search of both subject headings and keywords was constructed in Ovid MEDLINE, Ovid Embase, Ovid PsycInfo, EBSCO Social Services Abstracts and Web of Science Core Collection (supplementary eMethods 1, available at https://dx.doi.org/10.1192/bjo.2022.599). The final search was run on 17 July 2020, with no limits placed on date or country of origin. All search results were imported into Covidence software (www.covidence.org) and independently screened by title/abstract and subsequently full text by two reviewers (C.L., M.L.); conflicts were resolved by a third reviewer (Y.S.). From a total of 3409 original search results, 25 studies were selected for inclusion (Fig. 1). Following the later stages of the project (qualitative analysis, preliminary framework development, stakeholder analysis and final framework development), a second review of articles published between 17 July 2020 and 1 November 2021 was conducted to determine whether studies that were published after the initial search had uncovered new themes of personal recovery. This search identified an additional 721 articles, from which 4 articles were extracted using the same review method as the initial search (papers 26–9 in supplementary eTable 1). Independent review of these articles by three reviewers (C.L., S.H., M.L.) did not discover novel codes/themes, which suggests that our original search/thematic analysis had reached saturation.

### Eligibility criteria

Articles were eligible for inclusion if they met the following four criteria: (a) contains a conceptualisation of personal recovery from a suicidal episode as opposed to modelling predictors related to clinical recovery, such as symptom remission, decrease in suicide attempts or restoration of functioning from suicidal ideation/behaviour; (b) presents an original model, theory or framework of personal recovery or a modification or expansion of a previous model or framework; (c) conceptualisation is based on primary research involving quantitative or qualitative data with more than two participants (to restrict our review to empirically based research articles rather than case studies) or secondary research synthesising primary research; (d) full text is available in printed or downloadable form. Articles were excluded if they focused solely on clinical recovery, were not based on patients with an acute suicidal experience (either hospital admission due to suicide risk or suicide attempt with/or without hospital admission), were not peer reviewed (e.g. dissertations and doctoral theses) or were unavailable in English.

### Data analysis

#### Critical appraisal

After eligible articles were identified, two raters (C.L., M.L.) independently used Clark's guidelines on relevancy, appropriateness, transparency and soundness (RATS)^18^ and the Checklist for Analytical Cross-Sectional Studies (CACSS)^19^ to assess the quality of qualitative and quantitative articles respectively. Disagreements were resolved through consultation with a third rater (Y.S.).

#### Data extraction

Qualitative analysis of included article content was conducted by applying the rigorous and accelerated data reduction (RADaR) technique, a rapid matrix-based content analysis method used to reduce and organise raw qualitative data.^20^ First, four researchers (Y.S., C.L., M.L., C.R.) independently reviewed the 25 included studies. This review, and the eventual coding process, included the participants’ quotes as well as the manuscripts’ results and discussion sections. They then met to discuss emerging patterns and reduce the length of each document to include only portions most relevant to the research question. This cycle was repeated three times to continuously reduce the text.

#### Open coding and iterative thematic analysis

Four researchers (Y.S., C.L., M.L., C.R.) open coded 50% of the data, resulting in all data being double-coded. Coding was performed using Taguette, a qualitative data analysis software.^21^ Following the open-coding stage, a constant comparative method was used, in which we iteratively reviewed the resulting codes to eliminate infrequently occurring codes (<5 occurrences) and combine thematically similar codes.^22^ This constant comparative method was used to develop a parsimonious list of codes. An iterative thematic analysis was applied to organise the final list of codes into a preliminary model with overarching themes and related subthemes.

#### Stakeholder feedback

In May 2021, we contacted and sought feedback from three research centres and 46 individuals with expertise in recovery and/or suicide research and/or lived experience of suicide ideation, behaviour and/or attempts. The intent was to obtain feedback from a minimum of five experts from each of three domains: suicide treatment, recovery and lived experience of PASE recovery (individuals in recovery after a suicidal episode). Research experts were identified through referrals from prominent researchers, referrals from the three research centres and contacting authors of well-cited articles in the literature. Lived experience experts were individuals referred by clinicians, lived experience experts involved in the project (M.G., Y.S.) and lived experience experts who had already provided feedback. The response rate of those contacted was 59% (27/46).

Our stakeholder panel comprised individuals with expertise in research/clinical work on personal recovery (*n* = 22), suicide treatment/prevention (*n* = 13) or lived experience of PASE recovery (*n* = 7), with certain individuals having expertise in multiple domains (*n* = 8). Three research centres in the USA (one specialising in personal recovery, another in suicide prevention and the third in both) reviewed and provided feedback on the model. Respondents were located in the USA (*n* = 16), UK (*n* = 3), The Netherlands (*n* = 3), Australia (*n* = 2), Denmark (*n* = 1) and Canada (*n* = 1). Of the 27 individuals consulted, 17 were female and 10 were male. If an individual from a research centre was specifically quoted in the feedback, we counted them as a separate ‘individual expert’ beyond the general feedback received from the centre. The preliminary framework (Table 1) was presented, along with an overview of the rationale and method by which the framework was developed. We asked for general comments, themes we might be missing, better wording for the labels, clinical implications and any other feedback they had. A few (*n* = 3) were presented with the framework in an interview and thus provided feedback orally. The experts provided feedback in interviews and/or written responses in which they independently reviewed a written summary of the framework.

**Table 1:** Preliminary conceptual framework for the theoretical model of recovery after an acute suicidal episode

The seven recovery processes and their subcategories	Studies containing each process, *n* (%)
Connectedness	23 (92%)
Becoming more connected to others	14 (56%)
Open communication	12 (48%)
Improving relationships	8 (32%)
Secure, loving and meaningful relationships	7 (28%)
Support system	19 (76%)
Warm environment	16 (64%)
Therapy	15 (60%)
Spending time with others	11 (44%)
Support from others recovering from suicide	9 (36%)
Reintegrating into society	5 (20%)
Belongingness	21 (84%)
Feeling accepted	15 (60%)
Validation	13 (52%)
Feeling important to others	11 (44%)
Belonging	7 (28%)
Non-verbal presence and support	2 (8%)
Insight	21 (84%)
Self-understanding	14 (56%)
Self-awareness	11 (44%)
Thinking/reflection, including about painful experiences	4 (16%)
Insight	3 (12%)
Understanding through context of life history	1 (4%)
Gaining trust in humanity	3 (12%)
Hopefulness	15 (60%)
Positive thinking	1 (4%)
Evolving/moving past suicidality	21 (84%)
Understanding suicidality and recovery	10 (40%)
Comfortable discussing suicide	6 (24%)
Recognise effect of suicidality on others	4 (16%)
Decision to live	17 (68%)
Learning one's life has value	13 (52%)
Plan for the future	8 (32%)
Desire to live after a change in relationship with death	5 (20%)
Stage of ambivalence	2 (8%)
Identity growth	20 (80%)
Personal autonomy	15 (60%)
Identity	14 (56%)
Personal growth	8 (32%)
Developing empathy	6 (24%)
Separating identity from suicide	5 (20%)
Developing skills for living	19 (76%)
Developing useful skills	16 (64%)
Being productive/proactive	11 (44%)
Self-expression	5 (20%)
Self-care	5 (20%)
Verbal emotional catharsis	8 (32%)
Professional care	7 (28%)
Managing intense emotions	4 (16%)
Finding meaning	14 (56%)
Meaning in life	12 (48%)
Religion	8 (32%)
Spirituality	2 (8%)

Feedback was reviewed by a group of clinicians, researchers and lived experience experts to discuss how to properly address and incorporate it into the model. Then a group of the authors (Y.S., C.L., M.L., C.R., M.G.) incorporated it into the initial framework, resulting in a revised theoretical model (see Results section for additional information about our method of deciding how to incorporate feedback).

## Results

The scoping review resulted in 25 articles (Fig. 1): 19 qualitative and 6 quantitative. Studies were conducted in 10 countries, including 6 (24%) in the USA, 5 (20%) in Canada, 5 (20%) in Taiwan and 3 (12%) in the UK. Sample sizes ranged from 4 to 149 (mean 33; see supplementary eTable 1 for additional details). Of the 25 articles, 15 (60%) examined male and female participants, 2 (8%) examined only male participants, 2 (8%) examined only female participants and 6 (24%) did not specify whether participants were male or female. Our review encompassed a wide age range, from adolescence to geriatric samples. Although the studies in our review represented all these ages, the majority were of adults, with only 4 (16%) focusing on adolescents and 3 (12%) on the elderly. See Supplementary eTable 1 for article information and a brief summary of pertinent findings.

In the critical appraisal phase, RATS scores among the 19 qualitative studies ranged from 5 to 18 (mean 15; s.d. = 3.01). Twelve studies earned a score exceeding 14, indicating high quality. Among the quantitative studies, all CACSS scores exceeded 4, meeting the requirements for methodological rigour. A subset of articles with higher-quality rankings (RATS scores exceeding 14) were used for the first round of coding, as initial codes can have increased influence.^4^

Using the RADaR technique, 12 themes emerged from the initial 149 codes (see supplementary eMethods 2), which were organised into 7 processes to form the preliminary conceptual framework: connectedness, belongingness, insight, evolving/moving past suicidality, identity growth, developing skills for living and finding meaning (see Table 1 for preliminary framework subcategories and their prevalence in the literature).

To evaluate and improve the preliminary conceptual framework, it was shared with a stakeholder expert panel. All written and verbal feedback was thematically organised and its merits and applications were discussed by the research team (see Method section for additional information and supplementary eTable 1 for participant quotes). Thematic organisation of the responses was as follows: structural (combining overlapping areas of the model, e.g. connectedness and belonging; identifying underrepresented areas, e.g. peer support, the role of medication, self-compassion, empowerment and autonomy), language (sensitivity, e.g. some experts promoted the term ‘insight’ and ‘growing beyond the suicidal identity’ but others disagreed; clarity, i.e. selection of language matching individuals’ lived experience after an acute suicide episode) and validity (recovery processes that seemed in line with the experts’ clinical and/or lived experience). To move towards building a co-developed model, we gave particular weight to suggestions from individuals with lived experience. For example, one individual highlighted the difference between ‘feeling connected’ and ‘growing connected’, based on his experience of deepening pre-existing connections versus forming new connections. In line with this, we included ‘growing connectedness’ in the final model. The affirmative responses from the experts regarding the preliminary conceptual framework served as a validity check. Therefore, given the overall agreement of the experts on the validity of the preliminary framework, as well as the weight of the preliminary qualitative evidence derived from the empirical data, we were careful to integrate feedback into the final model in a manner that did not radically alter the preliminary framework.^1^

### The final seven categories

Following this revision stage, the model identified seven categories. Each category contains multiple subcategories organised by higher-order construct. Category labels were constructed by the research team by first developing preliminary labels to encapsulate each category and then developing an appropriate acronym. This required slight modification of the category labels to match a recognisable acronym. We selected an acronym that would allow us to use category labels that would not veer from the meaning of the preliminary label. For example, ‘optimising identity’ was considered a justified modification of ‘identity growth’, as it did not alter the meaning of the label and fit the acronym ‘COURAGE’. Ultimately, the final model included seven processes, with subcategories: choosing life, optimising identity, understanding oneself, rediscovering meaning, acceptance, growing connectedness and empowerment (giving the acronym COURAGE; see Appendix for the individual processes’ components; see supplementary eTable 2 for selected quotes relating to the individual processes).

#### Choosing life

Individuals make a cognitive and emotional decision to live, enabling an increase in interest in life and hopefulness. In turn, this can further the recovery process by helping them regain a desire to live and begin investing in life by planning for the future.

#### Optimising identity

The process of developing a sense of oneself as a valued individual with a coherent life story. For many, this process includes developing self-confidence, self-esteem, a clearer life role and a ‘post-suicidal’ identity in which the suicidal episode itself is seen as a source of personal growth.

#### Understanding oneself

The process of developing an understanding of oneself through reflection on one's life history, emotional reactions, behaviours, strengths and weaknesses. This process often includes learning about one's unique pattern of developing increased suicidality and moving towards personal recovery.

#### Rediscovering meaning

Discovering purpose and meaning in one's life enhances future-oriented beliefs and builds psychological resilience. For many, engaging in religion and/or spirituality provides a sense of community and higher purpose.

#### Acceptance

This process includes feeling accepted by others and accepting one's internal contradictions, pain and misalignment with others. Feelings of acceptance often emerge in the context of a safe environment to securely discuss negative thoughts and experiences.

#### Growing connectedness

Quality relationships with family, friends and community can lead to decreased loneliness and an increase in belonging, feeling valued, gaining faith in humanity and reintegration into society. In particular, support from others on a similar recovery journey helps engender a deeper sense of reintegration, belonging and connection.

#### Empowerment

The dual development of internally focused skills (e.g. self-expression, self-compassion and emotion regulation) and externally focused skills (e.g. empathy, hobbies and career-oriented abilities) generates productivity, self-efficacy, agency and personal responsibility. This process often includes developing knowledge and courage to seek and accept professional help.

### Individuals’ recovery journeys

The COURAGE processes may occur simultaneously or in any order. Overall, in many of the studies (*n* = 7), the personal recovery journey after a suicide attempt was described using variations of ‘multidimensional’, ‘non-linear’ and ‘complex’ – depicting a slow, gradual process of personal growth where the individual fluctuates between improvement and the occasional setback.

## Discussion

The results of this multistage search to identify the underlying components of recovery after an acute suicidal episode led to the seven COURAGE processes. Taken in a general sense, these seven processes span the scope of human life – what it is to live, grow and find meaning and purpose. Although these processes appear to reflect fundamental and universal human experiences, it is important to consider the inherent paradox of creating a standardised multicomponent model to describe a complex, individualistic journey. We do not expect every recovery journey to align exactly with our proposed model, nor do we assume that all individuals require growth in every COURAGE process. However, our findings, including the prevalence of these processes across the studies we reviewed, suggest that many individuals in PASE recovery will experience most, if not all of the COURAGE processes.

### The COURAGE model in relation to previous recovery models

As expected, there are many similarities between COURAGE and other mental health recovery models. SAMHSA's description of personal recovery as ‘a process of change through which individuals improve their health and wellness, live a self-directed life, and strive to reach their full potential’^3^ aligns well with the overall COURAGE framework and, in particular, with the processes ‘optimising identity’ and ‘empowerment’. However, unlike SAMHSA's model, COURAGE has less emphasis on improving health/wellness.

COURAGE also overlaps with the CHIME model of personal recovery after general mental illness, with overlap between their domains. Although subtle distinctions in emphasis exist, both models share some processes related to connection, identity, meaning and empowerment. However, some COURAGE processes are unique to the PASE personal recovery framework. For instance, ‘understanding oneself’ and ‘acceptance’ are not major components of the CHIME process, but they play a critical role in PASE recovery. Additionally, the idea of ‘choosing life’, while perhaps a subtle implication in CHIME, is a focal point in COURAGE. This is critical in the context of suicidality, in which individuals need to consistently reinforce this choice and, as one lived experience expert put it, ‘continuing to choose life again and again’. Similarly, ‘understanding oneself’ is particularly relevant for the awareness that is needed to identify reasons for wanting to end one's life. As one recovery expert with lived experience we consulted wrote, ‘The sense I get is that folks who have tried to take their life need to affirm life and the imperfect world. They need to restore their relationship to an imperfect world and imperfect humanity’. It appears that they need to engage in accepting the problems inherent in their environment, as well as their own flaws, which they may have viewed as unmanageable.

Even in the domains that overlap (between COURAGE and CHIME), there are important distinctions. For instance, although both include ‘empowerment’, the CHIME model's subcategories emphasise personal responsibility, strengths and control over life, whereas COURAGE finely tunes this in PASE recovery to focus on particular empowerment-related skills such as self-expression and career-oriented abilities. Additionally, although both CHIME and COURAGE have identity-related processes, CHIME emphasises rebuilding a positive sense of identity and overcoming stigma, whereas COURAGE includes improved self-perception but de-emphasises overcoming stigma and includes a wider array of identity subcomponents, such as life role clarity, narrative identity, post-suicidal identity development, and self-confidence and esteem.

Relatedly, it is important to note that we selected the acronym COURAGE as the letters matched the seven processes identified but the trait of courage is not itself one of the processes. However, the research team considered it an apt acronym because, for many individuals, personal recovery, striving to grow and working to overcome internal and societal challenges can at times require and be an act of courage.

### The COURAGE model in relation to suicide risk models

The COURAGE model describes a framework for personal recovery rather than suicide risk. However, it is likely that as individuals move forwards in personal recovery with richer and more meaningful lives they will also have lower risk profiles. Therefore, it is worth considering possible similarities and differences between the focuses of COURAGE and suicide risk models. We considered the interpersonal theory of suicide, which outlines three factors: thwarted belongingness, perceived burdensomeness and acquired capability.^23^ There are intriguing similarities between the interpersonal theory of suicide and the first of the COURAGE processes, ‘growing connectedness’. In particular, the subcomponent ‘belonging’ resembles Joiner's concept of ‘thwarted belongingness’.^23^ However, although the subcomponents ‘feeling valued’ and ‘becoming/feeling more connected to others’ may be related to Joiner's concept of ‘perceived burdensomeness’, there is an important distinction. Feeling valued and increasing connection with others is not just the absence of perceived burdensomeness but the positive addition of rich, meaningful interpersonal connections and value judgements about oneself from others. Furthermore, unlike Joiner's model, COURAGE does not focus on the development of suicidal ideation or behaviour. Rather, PASE recovery relates more to underlying issues related to personal recovery that lead a person away from a suicidal mindset – concerns about the meaning of life, identity, purpose and one's place among others. As expected, COURAGE is not a suicide risk model and, although progressing through COURAGE may be associated with decreased future suicidal behaviour, that is not its focus.

### Purpose and utility of the COURAGE model

We view the COURAGE model as an aid to the personal and clinical support of individuals after an acute suicidal episode. PASE recovery is highly complex; the COURAGE processes overlap, interrelate with one another and extend throughout the lifespan. Individuals who had attempted suicide 50 years before said that these processes remain deeply relevant to their life. Furthermore, our review of the literature and the comments of the lived experience experts/stakeholders we surveyed lead us to understand the COURAGE processes as having complementary relationships, in which development in one process may lead to change in others. In addition, these sources clarified for us that the seven COURAGE processes are not universal nor ordinal, as the PASE recovery experience can be idiosyncratic and non-linear.^24^ Unlike a simple direct path, an individual's recovery journey after an acute suicidal episode has been characterised in the literature we surveyed as one marked by twists and turns, with different aspects of living life and recovery interacting in complicated ways.^25,26^ To add yet another layer of complexity, suicidality and recovery are not binary states, as one can continue to experience suicidal ideation as part of one's recovery.^27^ With regard to this phenomenon, expert feedback stressed the chronic nature of suicidality *during* recovery. One expert compared suicidality during recovery with a ‘canary in the mine’, indicating a need for ‘self-care, boundaries, balance, etc.’. Another expert described PASE recovery as ‘learning to live a meaningful and connected life whilst desiring death’, noting how suicidality can persist throughout the recovery process.

In line with these complexities, our purpose in operationalising PASE personal recovery through the COURAGE model is to provide a guide for the individual, researcher, therapist, caregiver, family member or friend during this journey. Owing to the complexity of PASE recovery, it can be difficult to understand the challenges individuals face at each part of the journey. The COURAGE model seeks to help clarify the domains which individuals view as beneficial to the recovery journey with the intention of helping our understanding of their personal and clinical needs. In addition, the seven processes can be used to develop a rich assessment of where individuals are in their recovery and what their personal needs may be. This model, and instruments based on it, can be used in clinical research to better understand PASE recovery and the efficacy of PASE-focused interventions. Finally, many treatments currently available for those experiencing suicidality appear to have limited fit with COURAGE. Most treatments focus on preventing or alleviating the acute suicidal stage or suicide attempt,^28^ and those that do focus on the post-acute stage focus on only a few of COURAGE's processes. For example, a recently developed identity- and life story-focused intervention piloted in the James J. Peters Veterans Affairs Medical Center addressed multiple COURAGE processes, including optimising identity and rediscovering meaning, but did not adequately address growing connectedness.^29^ Our hope is that the COURAGE model will help in the development of PASE recovery treatments addressing all seven processes. Each domain can be assessed for cognitive, behavioural and emotional characteristics, which can help inform the selection of cognitive and behavioural techniques that are most likely to be effective. Growth in the seven recovery processes can be facilitated through repurposing established and/or developing novel techniques and the therapeutic relationship itself by modelling how to form meaningful connections (e.g. ‘growing connectedness’).

There may also be a need for creating interventions that deal with PASE recovery in a more holistic fashion, taking into account the individual's entire life history and developing a higher-order structure within which the processes can be encompassed. Such interventions would require a greater understanding of the complex interactions between the different domains of the individual's life using a higher-order structure as a guide. Additionally, within a recovery orientation, these and other interventions need not be – and should not be – limited to traditional psychiatric and psychological care but should also include peer support, sponsors and other recovery-aligned techniques. Finally, it should be noted that within a recovery orientation, it may be necessary for currently suicidal individuals to first receive medical treatment to allow for later engagement in the psychotherapeutic process, which would otherwise be hindered by comorbid severe mental disorders.

### Limitations and future directions

This study developed a robust theoretical model that will require empirical testing using validated measures for coherence, reliability and validity. Furthermore, the current COURAGE model is largely a theoretical psychological model. Despite our proposal that it be used to guide peers and family support in addition to traditional psychotherapy care, its framework needs to be translated into applied settings through further development. In line with this research programme, future research would benefit from the development of a psychometrically valid PASE recovery scale using the COURAGE model. A related but more profound limitation lies within our research aim. We sought to crystallise recovery principles into a unified model. However, this runs the risk of eliminating the nuance of the individual PASE recovery experience within a set of streamlined categories. Each COURAGE process represents the complex narrative of many individuals and, consequently, the labels selected cannot account for their diverse experiences. This complexity must be recognised when supporting individuals after an acute suicidal episode. Although our current sample includes studies from more than ten countries spanning six continents, data from additional diverse and cross-cultural samples would assist in the development of a universal model of PASE recovery. Additionally, the studies we found did not include sufficient data from particular populations that may have varying PASE recovery experiences, such as adolescents. To move closer to individualised models, future studies should explore the potentially varying experiences across age categories, genders, sexual identities, racial/ethnic/cultural identities and acuity of suicidal experiences (e.g. ideation, hospital admission prior to an attempt due to risk, single attempt or multiple attempts). Furthermore, the use of inductive thematic analysis to group our themes had an element of subjectivity, and other groupings would have been possible. Finally, as a large amount of the personal recovery theory and literature stems from a recovery frame developed for addiction,^30^ that frame is useful for thinking about how to implement the PASE recovery processes. For example, potentially viable methods such as peer-led groups and peer-based accountability would be helpful as a means of enhancing PASE recovery.

### Research and clinical implications

COURAGE has important implications for both researchers and clinicians to consider. Although understanding suicide risk and development is critical, there has been less of a focus among researchers and clinicians on the recovery process after the acute stage. As a unique population characterised in part by existential challenges related to their life purpose, individuals recovering from an acute suicidal episode stand to benefit from a meaningful framework of recovery in line with the COURAGE processes which goes beyond the traditional model of safety planning and risk reduction. Expanding our research and clinical focus beyond suicide risk assessment and development models of suicide trajectories may help enrich the lives of individuals who have suffered from suicidal ideation, intentions and behaviours, as well as those who continue to grapple with these difficulties. We hope that COURAGE will inform the reconceptualisation of assessment, research and clinical care of individuals recovering from an acute suicidal episode.

## Data Availability

Data availability is not applicable to this article as no new data were created or analysed in this study.

## Supplementary material

For supplementary material accompanying this paper visit http://doi.org/10.1192/bjo.2022.599. click here to view supplementary material

## Appendix

### COURAGE recovery processes and their subcategories

**Choosing life**

- Affirming life
- Changing relationship with death
- Hopefulness
- Learning one's life has value
- Moving through ambivalence
- Planning for the future
- Recognising effect of suicidality on others
- Recovery-oriented thinking

**Optimising identity**

- Improved self-perception
- Life role clarity
- Narrative identity
- Personal growth
- Post-suicidal identity development
- Self-confidence
- Self-esteem

**Understanding oneself**

- Self-awareness
- Thinking/reflection, including about painful experiences
- Understanding difficulties through context of life history
- Understanding one's emotional reactions, behaviours, strengths and weaknesses
- Understanding suicidality/recovery

**Rediscovering meaning**

- Higher purpose
- Meaning in life
- Religion
- Spirituality
- Value identification

**Acceptance**

- Accepting internal contradictions and pain
- Accepting external stigma
- Comfortable discussing suicide
- Feeling accepted
- Non-verbal presence and support
- Sensitive caregivers
- Validation
- Warm and safe environment

**Growing connectedness**

- Becoming/feeling more connected to others
- Belonging
- Feeling valued
- Gaining trust in humanity
- Improving relationships
- Open communication
- Secure, loving, and meaningful relationships
- Support system - general and from others recovering from suicide

**Empowerment**

- Agency
- Developing life skills
- Gaining empathy
- Emotion regulation (eg., verbal emotional catharsis)
- Personal responsibility
- Productive/proactive
- Professional care
- Self-care
- Self-efficacy

The application of the model requires a personalised approach, as categories apply to people differently. Subcategories also have to be chosen on the basis of the respective individual's values, goals and cognitive, emotional and social networks.
