# Supplementary material for: Theoretical model of recovery following a suicidal episode (COURAGE): scoping review and narrative synthesis
Source: BJPsych Open. 2022 Nov 17;8(6):e200. doi: 10.1192/bjo.2022.599 (PMC9707512; doi:10.1192/bjo.2022.599)
Supplement: Supplementary file 1 [file bjosup.zip › S2056472422005993sup001.docx]

**Contributors**

Author YS designed the study and conducted the protocol, authors YS, CL, and MR undertook the scoping review on the articles organized by librarian SW, and authors YS, CL, MG, LD, MR, CR and SH developed the framework based on the scoping review. All authors contributed to the writing of the manuscript. All authors contributed to and have approved the final manuscript.
