# Supplementary material for: Theoretical model of recovery following a suicidal episode (COURAGE): scoping review and narrative synthesis
Source: BJPsych Open. 2022 Nov 17;8(6):e200. doi: 10.1192/bjo.2022.599 (PMC9707512; doi:10.1192/bjo.2022.599)
Supplement: Supplementary file 1 [file bjosup.zip › S2056472422005993sup002.docx]

Supplement

Items included:

(P. 2) eMethods1: Search strategy

(P. 4) eMethods2: Full list of initial codes

(P. 6) eTable1: Article tabulation

(P. 12) eTable2: COURAGE Quotes

eMethods1: Search strategy

**Search conducted on 7/17/2021:**

**Ovid Embase 1731 results on 7/17**

1. exp suicidal behavior/

2. suicid*.tw,kw.

3. 1 or 2

4. recovery.tw,kw.

5. (Connectedness or Hope or hopefulness or Identity or "Meaning in life" or empowerment or optimism).tw,kw.

6. (life adj3 meaning).tw,kw.

7. (((meaning or quality) adj3 life) or spirituality).tw,kw.

8. ((connect* or relationship* or support or supportive) adj3 (family or families or peer or communit*)).tw,kw.

9. (hope or optimism or aspiration*).tw,kw.

10. (identity or stigma).tw,kw.

11. (empowerment or (personal adj3 (strengths or strength or responsibility))).tw,kw.

12. (theor* or framework or model or dimension or paradigm or concept*).tw,kw.

13. 4 or 5 or 6 or 7 or 8 or 9 or 10 or 11

14. 3 and 12 and 13

**Ovid Medline 1232 results on 7/17**

1. exp Self-Injurious Behavior/

2. suicid*.tw,kf.

3. 1 or 2

4. recovery.tw,kf.

5. (Connectedness or Hope or hopefulness or Identity or "Meaning in life" or empowerment or optimism).tw,kf.

6. (life adj3 meaning).tw,kf.

7. (((meaning or quality) adj3 life) or spirituality).tw,kf.

8. ((connect* or relationship* or support or supportive) adj3 (family or families or peer or communit*)).tw,kf.

9. (hope or optimism or aspiration*).tw,kf.

10. (identity or stigma).tw,kf.

11. (empowerment or (personal adj3 (strengths or strength or responsibility))).tw,kf.

12. (theor* or framework or model or dimension or paradigm or concept*).tw,kf.

13. 4 or 5 or 6 or 7 or 8 or 9 or 10 or 11

14. 3 and 12 and 13

**Ovid PsycINFO 1182 results on 7/17**

1. exp suicide/ or exp suicidal ideation/ or exp suicide prevention/ or exp suicidology/

2. suicid*.ti.

3. 1 or 2

4. recovery.mp.

5. (Connectedness or Hope or hopefulness or Identity or "Meaning in life" or empowerment or optimism).mp.

6. (life adj3 meaning).mp.

7. (((meaning or quality) adj3 life) or spirituality).mp.

8. ((connect* or relationship* or support or supportive) adj3 (family or families or peer or communit*)).mp.

9. (hope or optimism or aspiration*).mp.

10. (identity or stigma).mp.

11. (empowerment or (personal adj3 (strengths or strength or responsibility))).mp.

12. (theor* or framework or model or dimension or paradigm or concept*).mp.

13. 4 or 5 or 6 or 7 or 8 or 9 or 10 or 11

14. 3 and 12 and 13

**Web of Science Core Collection 1007 results on 7/17**

TI=(suicid*)

AND

TS=

(recovery) OR

(Connectedness or Hope or hopefulness or Identity or "Meaning in life" or empowerment or optimism)

(life NEAR/3 meaning)

(((meaning or quality) NEAR/3 life) or spirituality) OR

((connect* or relationship* or support or supportive) NEAR/3 (family or families or peer or communit*)) OR

(hope or optimism or aspiration*) OR

(identity or stigma) OR

(empowerment or (personal NEAR/3 (strengths or strength or responsibility)))

AND

TS=(theor* or framework or model or dimension or paradigm or concept*)

**Social Services Abstracts 242 results on 7/17**

suicid*

AND

(Recovery OR (Connectedness or Hope or hopefulness or Identity or "Meaning in life" or empowerment or optimism) OR (((meaning or quality) NEAR/3 life) or spirituality) OR ((connect* or relationship* or support or supportive) NEAR/3 (family or families or peer or communit*)) OR (hope or optimism or aspiration*) OR (identity or stigma) OR (empowerment or (personal NEAR/3 (strengths or strength or responsibility))))

AND

(theor* or framework or model or dimension or paradigm or concept*)

**Search conducted on 2/7/2022:**

**Medline- 2802 results on 2/1**

1. (Treatment outcome* or Engagement or Improvement or Treatment effect*or retention).mp.

2. exp Professional-Patient Relations/

3. ((Match or characteristics or factors or alliance or relationship or Preference) adj8 (psychotherapist* or subject* or counsel* or participant* or patient* or client*)).mp.

4. therapeutic relationship.mp.

5. therapeutic alliance.mp.

6. 2 or 3 or 4 or 5

7. exp Psychotherapy/

8. Psychotherapy.mp.

9. Psychotherapist.mp.

10. 7 or 8 or 9

11. (sampl* or participant* or data analys* or subject* or quantitative or qualitative or cohort or regression or random* or comparative).mp.

12. exp study characteristics/

13. 11 or 12

14. 1 and 6 and 10 and 13

**PsycINFO- 1853 results on 2/1**

1. (Treatment outcome* or Engagement or Improvement or Treatment effect*or retention).mp.

2. exp Therapeutic Alliance/

3. ((Match or characteristics or factors or alliance or relationship or Preference) adj8 (psychotherapist* or subject* or counsel* or participant* or patient* or client*)).mp.

4. therapeutic relationship.mp.

5. therapeutic alliance.mp.

6. exp Psychotherapy/

7. Psychotherapy.mp.

8. Psychotherapist.mp.

9. (sampl* or participant* or data analys* or subject* or quantitative or qualitative or cohort or regression or random* or comparative).mp.

10. exp psychotherapists/

11. exp therapist characteristics/

12. 2 or 3 or 4 or 5 or 11

13. 6 or 7 or 8 or 10

14. 1 and 9 and 12 and 13

**Web of Science- 1076 results on 2/1**

TS=(“Treatment outcome*” or Engagement or Improvement or “Treatment effect*” or retention)

TS=((Match or characteristics or factors or alliance or relationship or Preference) NEAR/8 (psychotherapist* or subject* or counsel* or participant* or patient* or client*))

TS=(“therapeutic relationship”)

TS=(“therapeutic alliance”)

TS=(Psychotherapy)

TS=(Psychotherapist*)

TS=(sampl* or participant* or “data analys*” or subject* or quantitative or qualitative or cohort or regression or random* or comparative)

eMethods2: Full list of initial codes (*n* = 149).

Accepting oneself/one's experiences, Agency, Asking for help, Attending Therapy, Avoid rumination, Awareness of choices, Awareness of emotions, Becoming empathetic, Being Listened to, Being proactive, Being Productive, Being with others, Belonging, Building emotional intelligence, Challenge destructive behaviors, Change in relationship with death, Change in self-perception, Changes in relationships, Choosing life, Collaborative care, Comfortable discussing suicide, Communication with others, Confront painful experiences, Connected to others, Connection to peers, Considering suicide's impact on others, Control, Coping skills, Creative Expression, Decision to live, Depth of interpersonal relationships, Desire to live, Developing personal responsibility, Developing self-esteem, Discussing emotions, Distancing from family, Distraction from suffering, Education about suicide/recovery, Emotional connection with others, Encouraging and supporting beliefs, Ensuring safety of patient, Establishing life goals/purpose, Exercise, Existential growth, Family Reputation, Feedback and guidance, Feeling Accepted, Feeling cared for, Feeling important to others, Feeling listened to, Feeling loved, Feeling Needed, Feeling Respected by Others, Flexible/Sensitive to Client Needs, Gaining trust in humanity, Having structure/routine, Hobbies, How others perceive them, Human warmth, Identity, Improve confidence, Improve mood, Improve outlook, Improving Functionality, Increasing Optimism, decreasing pessimism, Indirect Therapy, Insight, Instilling hope, Internal change, Judgement-free support, Learning about their situation, Learning Communication Skills, Learning how to live, Learning skills/tools for recovery, Learning their life has value, Leaving harmful relationships, Making safer choices, Managing intense emotions, Meaning in Life, Medical care, Medicine, Multidimensional process, Negative view of death, Non-linear path, Non-verbal presence and support, Open communication, Open-minded thinking, Opportunities created by suicide, Personal Autonomy, Personal efficacy, Personal growth, Pets, Plan for the future, Positive change, Positive thinking, Post-Traumatic Growth, Problem solving, Professional care, Realizing others care, Recognize effect of suicidality on others, Recognizing difficulty in recovery, Reconnecting with others, Reconnection with the self, Regaining control, Reintegrating into society, Relationship with God, Relationships, Relief, Religion, Resolution of despair, Respecting Human Rights, Safe Space to Heal, Secure relationships, Self awareness, Self Expression, Self reflection, Self-care, Self-development, Self-Efficacy, Self-understanding, Separating identity from suicide, Shifting focus, Showing compassion, Showing empathy, Slow and difficult path, Slowly reaching out to social network, Spending time, Spirituality, Spouse/family support, Stage of ambivalence, Support from others recovering from suicide, Support System, Support with Medical Care, Supporting others, Taking care of self, Taking responsibility for family, Therapeutic relationship, Thinking/Reflection, Trusting others, Understanding Life History, Understanding suicidality, Understanding through context of life history, Unique journey, Validating relationship with death, Validation and feeling understood/seen, Valuing their relationships, Verbal emotional catharsis, Vulnerability, Warm environment

**eTable1: Preliminary conceptual framework**

| **The Seven Recovery Processes** | **Number (%) of studies containing each process** |
| --- | --- |
| **Connectedness**   - Becoming more connected to others - Open communication - Improving relationships - Secure, loving, and meaningful relationships   ------------------------------------------------------------------------------^a^   - Support System - Warm Environment - Therapy - Spending time with others - Support from others recovering from suicide - Reintegrating into society | **23 (92%)**  14 (56%)  12 (48%)  8 (32%)  7 (28%)  19 (76%)  16 (64%)  15 (60%)  11 (44%)  9 (36%)  5 (20%) |
| **Belongingness**   - Feeling accepted - Validation - Feeling important to others - Belonging - Non-verbal presence and support | **21 (84%)**  15 (60%)  13 (52%)  11 (44%)  7 (28%)  2 (8%) |
| **Insight**   - Self-understanding - Self-awareness - Thinking/reflection, including about painful experiences - Insight - Understanding through context of life history   -------------------------------------------------------------------------------   - Gaining trust in humanity - Hopefulness - Positive thinking | **21 (84%)**  14 (56%)  11 (44%)  4 (16%)  3 (12%)  1 (4%)  3 (12%)  15 (60%)  1 (4%) |
| **Evolving/Moving Past Suicidality**   - Understanding suicidality and recovery - Comfortable discussing suicide - Recognize effect of suicidality on others   -------------------------------------------------------------------------------   - Decision to live - Learning one’s life has value - Plan for the future - Desire to live after a change in relationship with death - Stage of ambivalence | **21 (84%)**  10 (40%)  6 (24%)  4 (16%)  17 (68%)  13 (52%)  8 (32%)  5 (20%)  2 (8%) |
| **Identity Growth**   - Personal autonomy - Identity - Personal growth - Developing empathy - Separating identity from suicide | **20 (80%)**  15 (60%)  14 (56%)  8 (32%)  6 (24%)  5 (20%) |
| **Developing Skills for Living**   - Developing useful skills - Being productive/proactive - Self-expression - Self-care   -------------------------------------------------------------------------------   - Verbal emotional catharsis - Professional care - Managing intense emotions | **19 (76%)**  16 (64%)  11 (44%)  5 (20%)  5 (20%)  8 (32%)  7 (28%)  4 (16%) |
| **Finding Meaning**   - Meaning in life - Religion - Spirituality | **14 (56%)**  12 (48%)  8 (32%)  2 (8%) |

^a^Dotted lines indicate the beginning of a new group of categories within the respective theme.

**eTable1: Article tabulation**

| **ID** | **Title** | **Journal Published** | **Year** | **Location** | **Method** | **Rating** | **Brief Summary of Pertinent Findings** |
| --- | --- | --- | --- | --- | --- | --- | --- |
| 1 | A Grounded Theory Study of Action/Interaction Strategies Used When Taiwanese Families Provide Care for Formerly Suicidal Patients | Public health nursing | 2009 | Taiwan | QL (Qualitative) | 14 | Action/interaction strategies for care of relatives: 1. guarding the person day and night 2. maintaining the activities of daily living 3. creating a nurturing environment |
| 2 | A modified grounded theory study of how psychiatric nurses work with suicidal people | International journal of nursing studies | 2006 | United Kingdom | QL | 15 | Key process of recovery: “re-connecting the person with humanity” through a 3-stage process: reflecting an image of humanity, guiding the individual back to humanity, and learning to live. |
| 3 | A suicidal recovery theory to guide individuals on their healing and recovering process following a suicide attempt | Journal of advanced nursing | 2013 | Taiwan | QL | 16 | Core category: Striving to accept the value of self-in-existence. Other key categories linked to this core category: becoming flexible and open-minded, re-building a positive sense of self, and endeavoring to live a peaceful and contented life. |
| 4 | A theory for the nursing care of patients at risk of suicide | Journal of advanced nursing | 2006 | Taiwan | QL | 14 | Core category: safe and compassionate care via the channel of the therapeutic relationship, other key categories: providing holistic assessments; providing protection; providing basic care; promoting healing through advanced care. |
| 5 | Being trapped in a circle: Life after a suicide attempt in Taiwan | Journal of Transcultural Nursing | 2001 | Taiwan | QL | 12 | Participants suffered from “being trapped in a circle” through three main avenues conceptualized under a broader theme of alienation versus connectedness. |
| 6 | Changes in Attitudes towards Life and Death during Recovery from a Serious Suicide Attempt | OMEGA-Journal of Death and Dying | 1971 | USA | QN (Quantitative) | 5 | Death grows more abhorrent to the suicide attempter as they begin to recover. |
| 7 | Content analysis of previously suicidal college students' experiences | Death studies | 1998 | USA | QN | 5 | Participants most often relied on family and friends during a suicidal episode and should be careful in choosing whom to confide in. |
| 8 | Counseling for suicide: Client perspectives | Journal of Counseling & Development | 2002 | Canada | QL | 15 | Therapeutic processes: (a) validating relationships, (b) working with emotions, and (c) developing a new identity. |
| 9 | Disconnection, reconnection and autonomy: four young South African men's experience of attempting suicide | Journal of youth studies | 2017 | South Africa | QL | 17 | Major themes: (1) ‘Turning away from others and self’, disconnecting from close others and from self, prior to attempting suicide as an act of autonomy; (2) ‘Returning to self and others’, re-connecting with others and gaining a healthy sense of autonomy. |
| 10 | Experiences that inspire hope: Perspectives of suicidal patients | Nursing ethics | 2018 | Norway | QL | 16 | Themes: Experiencing hope through (1) encounters, (2) atmosphere of wisdom and (3) taking back responsibility. |
| 11 | Factors that contribute to psychological resilience to suicidal thoughts and behaviours in people with schizophrenia diagnoses: qualitative study | BJPsych open | 2019 | England | QL | 14 | Psychological resilience to suicidal thoughts and behaviours: (a) understanding experiences (b) active behaviours (c) relationship dynamics |
| 12 | Follow-up study of patients with attempted suicide | International journal of social psychiatry | 1988 | Germany | QL | 5 | An important focus of therapy should be the strengthening of active strategies for coping with interpersonal problems.  An attempt should be made to initiate a strengthening of the  patient’s labile self-esteem. |
| 13 | Giving up or finding a solution? The experience of attempted suicide in later life | Aging and Mental Health | 2006 | England | QL | 16 | Broad themes: Struggle (before and after the attempt, and in relation to growing older), Control, and Visibility (feeling invisible or disconnected from others and either becoming more or less connected afterward). |
| **ID** | **Title** | **Journal Published** | **Year** | **Location** | **Method** | **Rating** | **Summary of Findings** |
| 14 | Healing and recovering after a suicide attempt: a grounded theory study | Journal of clinical nursing | 2014 | Taiwan | QL | 13 | Five phases: (1) self-awareness (2) the interrelatedness of life (3) the cyclical nature of human emotions (4) adjustment (5) acceptance. While each phase might follow the preceding phase, it is not a linear process. |
| 15 | Healing from suicide: adolescent perceptions of attachment relationships | British Journal of Guidance & Counselling | 2007 | Canada | QL | 17 | Participants consistently reported the creation of a secure attachment as being pivotal to healing from suicidal feelings. |
| 16 | ‘‘I think I am worth it. I can give up committing suicide’’: Pathways to recovery for Chinese-Canadian women with a history of suicidal behaviour | Transcultural psychiatry | 2019 | Canada | QL | 14 | “Survival’’ recovery in the short term and ‘‘thriving’’ recovery in the long term, with survival strategies extending into the thriving phase of recovery during their complex path to it. |
| 17 | Is it possible to overcome suicidal ideation and suicide attempts? A study of the elderly | Ciencia & Saude Coletiva | 2015 | Brazil | QL | 17 | Five centers of meaning: religiosity and religious practices; social and family support; the support of health services; contact with pets; and the recovery of the autonomy to manage their own lives. |
| 18 | Social Support Mediates the Association between Disclosure of Suicide Attempt and Depression, Perceived Burdensomeness, and Thwarted Belongingness | Suicide and Life‐Threatening Behavior | 2020 | USA | QN | 4 | It is important to consider social support when discussing disclosure of suicide attempt. |
| 19 | Spiritual Well-Being and Depressive Symptoms in Female African American Suicide Attempters: Mediating Effects of Optimism and Pessimism | Psychology of Religion and Spirituality | 2014 | USA | QN | 4 | Spiritual well-being and depressive symptoms were mediated indirectly through both optimism and pessimism; greater religious and existential well-being was related to more optimism, and less pessimism and, in turn, to fewer depressive symptoms. |
| 20 | Suicidal adolescents: Helpful aspects of psychotherapy | Archives of Suicide Research | 2003 | Canada | QL | 17 | The findings suggest that participants found 5 aspects of therapy to be helpful: Enhanced Self-Understanding, Communication, Creative Expression, Therapeutic Relationship, and Therapeutic Strategies. |
| 21 | Survivors of Suicide Attempts Support Group: Preliminary Findings from an Open-Label Trial | Psychological services | 2018 | USA | QN | 5 | Participants’ use of their own experiences to help others (e.g., through empathic sharing and group problem solving), and the subsequent recognition that they have something to offer to other group members, may also reduce perceptions of being a burden. |
| 22 | Taking care of oneself by regaining control - a key to continue living four to five decades after a suicide attempt in severe depression | BMC psychiatry | 2017 | Sweden | QL | 18 | Three categories described the recovery process: ‘coming under professional care’, ‘experiencing relief in the personal situation', and ‘making a decision to continue living,’ with a core category, labeled ‘taking care of oneself by regaining control’. |
| 23 | Talking about suicide may not be enough: family reaction as a mediator between disclosure and interpersonal needs | Journal of Mental Health | 2017 | USA | QN | 5 | Higher rates of disclosure predict more positive family reactions, which in turn predict lower levels of thwarted belongingness and perceived burdensomeness. |
| 24 | The Perspectives of Young Adults on Recovery from Repeated Suicide-Related Behavior | Crisis: The Journal of Crisis Intervention and Suicide Prevention | 2009 | Canada | QL | 18 | The struggle to live is a process involving a fluid pathway moving between key elements: (a) living to die, (b) ambivalence and tipping/turning points, and (c) a process of recovery that included small steps or phases (pockets of recovery) toward life. |
| 25 | Young People's Pathways to Well-being Following a Suicide Attempt | International Journal of Mental Health Promotion | 2002 | New Zealand | QL | 18 | Useful techniques in the transitions towards attaining a sense of self-responsibility and increased agency: active help-seeking from professionals and peers, practical problem-solving abilities, thinking positively, and rediscovering a ‘will to live’. |
| 26 | The Male Experience of Suicide Attempts and Recovery: An Interpretative Phenomenological Analysis | International Journal of Environmental Research and Public Health | 2021 | United Kingdom | QL | 18 | Four themes were identified: (1) characteristics of attempt/volitional factors, (2) dealing with suicidal  thoughts and negative emotions, (3) aftermath and (4) protective factors. |
| 27 | The meaning of life after a suicide attempt | Archives of Psychiatric Nursing | 2022 | Brazil | QL | 18 | Three themes: “Searches in the affective, relational and spiritual field” (manifestations of affection, zeal, understanding and welcome and seemed to increase the link and commitment to life, as well as the openness to resignify it); “Life and the constancy of impermanence” (life as an alternation between weakness and strength, crisis and well-being, problems and overcoming); and “Discoveries and relationship with one's own self” (a self previously self-destructive could be resigned as capable of overcoming, resisting and developing resilience). |
| 28 | Protective factors for older suicide attempters: Finding reasons and experiences to live | Death studies | 2019 | Australia | QL | 18 | Between-case analysis identified four main categories: interpersonal relationships, meaningful activities and interests, community engagement, and involvement of mental health services staff. |
| 29 | You feel it was written about you: client acceptability of a group intervention for repeat suicide attempts | Journal of Mental Health | 2020 | Ireland | QL | 17 | Three interrelated response processes: connection with each  other and the model, taking control of life, and gaining a sense of value. |

eTable2: COURAGE Quotes

| Choosing life | “The attempt seemed to have mobilised some participants and their support systems, providing them with help that was not available beforehand, and in doing so gave them a renewed energy and a marked sharpening of focus and interest in life.” 14  “Re-discovery of a ‘will to live’ served as a signal of a transition from needing care and protection towards increasing self-responsibility and personal agency.” 30  “I suppose, I’m absolutely bloody terrified of dying like you know. I just don’t want to die and like I want to see my kids get older. I know I’m getting mushy but I want grandchildren and stuff like that and it’s like more reality to me now than it ever was I think. Just lately it’s like everything looks a bit brighter.” 30  “When I woke up, I realised that this suicide is a very painful thing… I had attempted once before. A light went on in my head and I then understood that …I don’t want to die… in fact, I’m really afraid of death.” 15 |
| --- | --- |
| Optimizing identity | “Co-constructing a new narrative that shifts the woman from the role of victim to the role of a strong person who has agency in her life.” 18  “The reflections and feedback helped this participant to see own patterns in thoughts and actions, but also own possibilities.” 11  “Participants reasoned that their suicidal thoughts did not necessarily define them as individuals but were perceived as aspects of life that they had to adapt to in the long term.” 12  “...struggle to minimise her identification with her ‘should’ side, that is, the identity which encourages her to act on her suicidal thoughts.” 30 |
| Understanding one’s self | “Understanding was based on individuals’ perceptions of their experiences and the different ways they made sense of these over time. The process of understanding leads to reconciliation to and acceptance of these experiences, and ultimately, finding purpose in life.” 12  “Many had kept their emotions ‘bottled up’ and come to realise that they needed to express them in their own unique way. Gaining this insight helped them cope with unstable emotions.” 15  “In addition to developing new coping mechanisms, participants focused on understanding suicidal behavior by making connections among their thoughts, feelings, and actions.” 25  “Being suicidal was framed as an attempt to resolve distressing personal issues within the context of minimal repertoire of coping strategies.” 25 |
| Rediscovering meaning | “Also associated with resilience and well-being, existential meaning and purpose may provide suicidal individuals with a sense of personal insight, growth, and life purpose.” 24  “From the perspective of the respondents, belonging to a religious group means feeling useful, to be alive, to have the opportunity to speak and to be heard, to share anxieties with others, to practise physical activities, and to exercise a sense of belonging and purpose in life.” 19  “The extent to which an individual engages with God or a higher power in a positive and satisfying manner, or has a sense of meaningfulness and purpose in their life, may influence their cognitive-emotional functioning including future-oriented beliefs.” 24  “It was not me who gave me life. It was him who gave it to me (referring to God). So it is he who has the right to take my life. So I have to wait until the time that he decides.” 19 |
| Acceptance | “‘…what you do is accept the fact that it [the suicidal thought] exists […] you just have it as an existing part of your mind.” 12  “Participants said that as they moved through the phases, they began to accept and embrace self, others and their current life situation.” 15  “Talk[ing] about their feelings, thoughts and experiences without any sense of judgment was a liberating and emancipatory experience.” 2  “However, all participants reported that they gained courage to connect with close others again when close others expressed that participants are loved, respected and form part of their lives.” 10 |
| Growing connectedness | “In the aftermath of their suicide attempt, participants were reminded that others care about them and that close relationships endow life with meaning.” 10  “After they attempted suicide, they realised the painful impact their attempt had on their children. Observing their anguish helped participants realise that they were important and that their life was precious. This was ‘the first time’ they had acknowledged that: “‘I’m important and my life’s important too’.” 15  “Many participants described the experience of support as facilitating a process of opening up to themselves in addition to others.” 16  “Paradoxically, participants reported that their suicide attempt allowed them to re-establish connection and find relatedness, which seems to have facilitated their recovery from suicidality.” 10 |
| Empowerment | “Now I have space to do my things, take care of my house, make my food the way I like it. This resumption of control of her life brings with it a sense of pleasure and the possibility of projection into the future.” 19  “Surviving their suicide attempt provided them with the courage to be themselves and instilled in them an increased sense of autonomy.” 10  “An increased sense of self-efficacy through learning new coping strategies contributed to a more positive self-identity.” 25  “Importance of problem solving as a means of enhancing the transition towards self-responsibility and resistance to future suicidal behaviours, a crucial component was developing an ability to address problems appropriately as they occurred.” 30 |
